# African families’ and caregivers’ experiences of raising a child with intellectual disability: A narrative synthesis of qualitative studies

**DOI:** 10.4102/ajod.v10i0.827

**Published:** 2021-04-30

**Authors:** Siyabulela Mkabile, Kathrine L. Garrun, Mary Shelton, Leslie Swartz

**Affiliations:** 1 Department of Psychology, Faculty of Arts and Social Sciences, Stellenbosch University, Stellenbosch, South Africa; 2 Department of Psychiatry and Mental Health, Faculty of Health Sciences, University of Cape Town, Cape Town, South Africa; 3 Faculty of Health Sciences, University of Cape Town, Cape Town, South Africa

**Keywords:** intellectual disability, children, families, Africa, caring, experience, culture, services

## Abstract

**Background:**

The prevalence of intellectual disability was high in Africa, particularly amongst low socio-economic communities. Despite this, there was limited literature on primary caregivers and parents of people with intellectual disabilities regarding their experience raising an individual with the condition, especially within the African context.

**Objectives:**

The aim of the current systematic review was to investigate experiences of caregivers and parents of children with intellectual disability in Africa.

**Method:**

We used strict eligibility criteria to identify suitable studies. We identified Medical Subject Headings (MeSH) terms and other keyword terms and, after conducting searches in electronic databases, identified articles that met the inclusion criteria for articles published between 1975 and the end of 2019.

**Results:**

164 articles were assessed for eligibility. Nine studies met the review’s criteria. Six major themes emerged: understanding of intellectual disability (ID), worries about the future, burden of care, lack of services, coping strategies and stigma and discrimination.

**Conclusion:**

Caregivers of children with intellectual disability in Africa faced substantial challenges. Current findings suggested that there was the need for both formal and alternative healthcare workers to work together towards an understanding and management of intellectual disability in Africa.

## Introduction

Despite the higher prevalence rate of intellectual disability (ID) in low- and middle-income countries (LMIC) compared with high-income countries (Maulik et al. 2011), there has been limited research in LMIC and in Africa specifically (Adnams 2010; Mckenzie, McConkey & Adnams 2013). A number of studies have reported that parents and caregivers of children with ID report negative experiences compared with those raising children without ID (Bristol, Gallagher & Schopler 1988; Dyson 1997; Hayes & Watson 2013; Lloyd & Hastings 2009; Olsson & Hwang 2001). Some studies reported that parents of children with ID may experience anxiety, post-traumatic stress disorder (PTSD) and even depression when told about the diagnosis of their children. Other studies have demonstrated that parents may experience high levels of stress during the caring process, especially when a child presents with a challenging behaviour (Hassall, Rose & McDonald 2005). This situation is exacerbated for parents and caregivers living in low-income countries, with parents reporting more severe levels of stress, severe sadness, family difficulties, financial difficulties, stigma, shame and discrimination (Azar & Badr 2010; McKenzie & McConkey 2016; Sen & Yurtsever 2007; Tilahun et al. 2016). Some studies have demonstrated the role played by culture and religious beliefs in shaping caregiver and parental experiences in caring for a child with ID. Whilst significant contributions and strides have been made in the literature on caregivers’ and parents’ experiences in various contexts, little is known about the experiences of black African caregivers and parents of children with ID in Africa, where specialised services for people with intellectual disability (PWID) and their families are limited or non-existent. A scoping review on services for children with disabilities in LMIC (Magnusson, Sweeney & Landry 2019) indicates the paucity of services and consequent impact on families. It is clear that access to rehabilitation services in Africa is a challenge (Morris et al. 2019).

For African caregivers and parents, experiences and outcomes of raising or caring for PWID are expected to be worse, in part because of the legacies of colonialism (and in some countries, apartheid), poverty and poor living conditions in Africa. In addition to other challenges, these families have been reported to have high rates of single parenthood, child-headed households, fatherlessness, alcohol abuse and domestic violence (Scior et al. 2015). In Southern Africa and other parts of the continent, the effects of migrant labour, where men have to leave their wives and children to seek employment, usually in the cities, have also significantly affected black African families. The displacement of family members during political struggles in a number of countries might have also contributed to the current functioning and structure of the African family system (Siwella 2011).

Most work in the field of ID in Africa has been conducted in South Africa. However, important publications from the continent at large do exist. Gona et al. (2015) write about the perceptions of professionals and parents on the causes and treatment options for autism in Kenya. The authors conducted a qualitative study in a multicultural context and found that, regardless of culture, participants held similar perceptions regarding the causes of, and treatment options for, autism. Similar to that what has been found in other contexts (McKenzie & McConkey 2016), in Kenya, caregiver perceptions about the causes of autism ranged from supernatural beliefs, such as evil spirits, witchcraft and curses, to biomedical causes related to infections, drug abuse, birth complications, malnutrition and hereditary conditions (Gona et al. 2015). Perceptions regarding treatment options encompassed both biomedical and traditional and spiritual methods of healing (Gona et al. 2015). Indeed, across the continent, it has been reported that it is common in African cultures to use both Western and traditional healing systems (Kromberg et al. 2008).

Whilst it has been suggested that, in different cultural contexts, negative reactions may be moderated by cultural support systems or may be exacerbated by cultural beliefs and taboos (Empson 2015; Serpell, Mariga & Harvey 1993), the evidence, either in favour of or against this view in urban settings, is sparse in urban African settings (Empson 2015). One country where African cultural beliefs were found to be associated with negative reactions in some instances, but promoted the social inclusion of people with disabilities in others, is Swaziland (Ndlovu 2016). A global review on stigma and awareness raising by Scior et al. (2015) reported that in LMIC, including countries in Africa, children and adults with ID continue to experience high levels of stigma and are denied many rights and freedoms enjoyed by people without ID. Scior et al. (2015) observed the invisibility of PWID:

[*I*]s accompanied by low expectations of people with intellectual disabilities, in many countries they are still widely viewed as incapable, unable to live independently or contribute to society. Respondents noted that in many parts of Africa and Asia, in Russia, and in some parts of South and Central America there is often still an active desire to segregate people with intellectual disabilities from society due to deep rooted prejudice or stigmatising beliefs about the causes of intellectual disability. (p. 4)

These studies indicate that cultural beliefs, as well as religious belief systems, may offer important contributions to our understanding of how people with ID are perceived in Africa.

When considering the South African context (the African country with the highest research output), specifically with regard to the question of disability, it becomes clear that, despite the fact that South Africa is an upper middle-income country, serious challenges resulting from lack of resources and inherent socio-economic inequalities continue to prevail in black African communities (Makiwane 2010). In addition, the majority of black Africans remain trapped in extreme poverty, with many still lacking access to basic resources and infrastructure (Pillay 2008). Furthermore, broader studies on mental health on the continent suggest that mental health services for children are extremely limited in some African countries (Yoder et al. 2016). These challenges further complicate and frustrate parental efforts to provide care and support for their child with ID (Ataguba, Akazili & McIntyre 2011). As such, raising a child in a black African family that experiences an overwhelming psychological reaction associated with discovering that the child has ID, in a context of widespread poverty and deprivation, may be complex (Mbazima 2016). Some studies on African families have reported strong traditional belief systems and it is important to understand how these beliefs are shaped or challenged by the birth of a child with ID. It is also important to understand how African families cope with the reported stigma associated with the birth of a child with ID (UNICEF 2012).

In light of the above discussion, it is imperative that we understand how the birth of a child with ID affects the family system in a complex low-income African context. In order to address this need, we conducted a narrative synthesis of qualitative studies on the subjective experiences of caregivers and parents of children with ID regarding their caregiving experiences in order to identify gaps in the literature regarding caregiver or parent and family experiences in Africa. We chose to focus on qualitative research owing to the paucity of information and the lack of validated quantitative instruments in the African context (Christianson et al. 2002). Qualitative research is likely to provide detailed in-depth descriptions on which further work can be based. Our primary aim in this article is to review what is known about the experience of being a family caregiver for a child with ID in Africa. We believe that the imbalance of knowledge between wealthier and less wealthy countries regarding disability and specifically caring for a child with disabilityrequires more careful and thoughtful consideration, especially given the fact that disability is more prevalent in low-income contexts (Swartz 2014; Swartz & Marchetti-Mercer 2018).

## Theoretical framework

To our knowledge, there is no review that has attempted to examine the experiences of caregivers and parents of children with ID in Africa. A review from Africa examined human rights of individuals with intellectual disabilities in South Africa. Although in their review (Swartz & Marchetti-Mercer 2018), they make a distinction between a social and a medical model of disability, the review does not apply a socio-ecological approach. In the current review, we examine the experiences of caregivers and parents of children with ID in Africa using the socio-ecological model (Bronfenbrenner 1992) in order to identify potential targets for change in the provision of ID services in Africa across various systems.

Evidence suggests that most caregivers and parents of children with ID report a number of negative experiences across all levels of care. Bronfenbrenner’s socio-ecological model is helpful in exploring experiences of various sectors of care and support (Bronfenbrenner 1992). In line with this framework, caregivers and parents may have both positive and negative experiences of caring for a child with ID through their day-to-day interactions with different levels of healthcare and other systems.

Bronfenbrenner (1992) describes the social context as characterised by five dynamic interdependent and interrelated systems. These are the micro-, meso-, exo-, macro- and chrono-systems. Our key interest in this review is on the micro-system at the family or household level, but as Bronfenbrenner (1992) observed individuals have continuous interactions across different levels of the social context. Individuals might be influenced by, or might have influence through their continuous interaction with various systems. In order to develop a nuanced understanding of the caregivers’ and parents’ experiences, it is important to gain a deeper insight into the types of individuals’ lived experiences, the level of the social context at which experiences occurred and the consequent impact.

Bronfenbrenner’s (1992) framework takes due account of both material and cultural factors and facilitates an understanding of caregivers’ experiences of ID and its management at various levels and within different healthcare systems (Swartz 1998). Although our own approach to the field is influenced by a contextual understanding of local explanatory models and practices (Mkabile & Swartz 2020), we did not select articles based on any specific theoretical orientation. Indeed, we show here that there is a paucity of research on our topic of interest; an aspect of our motivation for undertaking the review is to demonstrate the scant state of current knowledge in the area and to encourage further research.

## Research methods and design

### Search strategy

We followed the Preferred Reporting Items for Systematic Review and Meta-Analysis (PRISMA) guidelines to conduct our study. We searched Ebscohost, Pubmed, Web of Science and Scopus. We searched for studies from 1975 to 2019, a longer period than is common, in order to access as many articles as possible from what we perceived would be a sparse field. A shorter time frame might have severely limited the number of eligible studies (Meline 2006) on experiences of raising a child with ID in Africa. The search terms used are presented in Box 1.

BOX 1 Search by Medical Subject Headings (MeSH) terms.

- TOPIC: ‘Intellectual Disability’ OR ‘Developmental Disabilities’ OR ‘Neurodevelopmental Disorders’
- AND TOPIC: ‘Child’ OR ‘Infant’ OR ‘Adolescent’
- AND TOPIC: ‘Caregivers’ OR ‘Community Health Nursing’ OR ‘Community Mental Health Services’ OR ‘Family’ OR ‘Foster Home Care’ OR ‘Home Care Services’ OR ‘Home Health Nursing’ OR ‘Health Personnel’ OR ‘Human Rights’ OR ‘Human Rights Abuses’ OR ‘Nurses’ OR ‘Patient Care’ OR ‘Psychotherapy’ OR ‘Rehabilitation’ OR ‘School Teachers’ OR ‘Violence’
- AND TOPIC: ‘Health Knowledge, Attitudes, Practice’ OR ‘Health Literacy’ OR ‘Attitude to Health’ OR ‘Patient Acceptance of Health Care’ OR ‘Sense of Coherence’ OR ‘Comprehension’ OR ‘Mental processes’ OR ‘Hermeneutics’ OR ‘Complementary Therapies’ OR ‘Culture’ OR ‘Faith Healing’ OR ‘Health Services, Indigenous’ OR ‘Herbal Medicine’ OR ‘Integrative Medicine’ OR ‘Plants, Medicinal’ OR ‘Religion’ OR ‘Religious Personnel’
- AND TOPIC: ‘Africa’
- AND TOPIC: Not ‘African American’

We used Braun and Clarke’s (2006) thematic analysis for the synthesis of studies selected for review; thematic analysis was the dominant mode of analysis in the reviewed studies. Thematic synthesis, based on Braun and Clarke’s (2006) approach, was used to combine results from all nine studies on caregivers’ experiences of raising a child with ID in Africa. This was a three-stage process involving: (1) the initial line-by-line coding of the results of all nine studies, (2) organisation of codes to construct descriptive themes and (3) the development of analytical themes (Braun & Clarke 2006). Although there are few studies that have used this approach for systematic reviews (Thomas & Harden 2008), thematic analysis is flexible and uses an inductive approach, which allows for the generation of themes. Seven studies that we reviewed (Aldersey 2012; Aldersey, Turnbull & Turnbull 2014; Gona et al. 2011; Lamptey 2019; Masulani-Mwale et al. 2016; Nkhosi & Menon 2015; Ntswane & Van Rhyn 2007) used qualitative methods and two studies (Ajuwon & Brown 2012; Tilahun et al. 2016) used mixed methods with a substantial qualitative component, providing sufficient qualitative material for this synthesis.

### Screening and study selection

**FIGURE 1:**
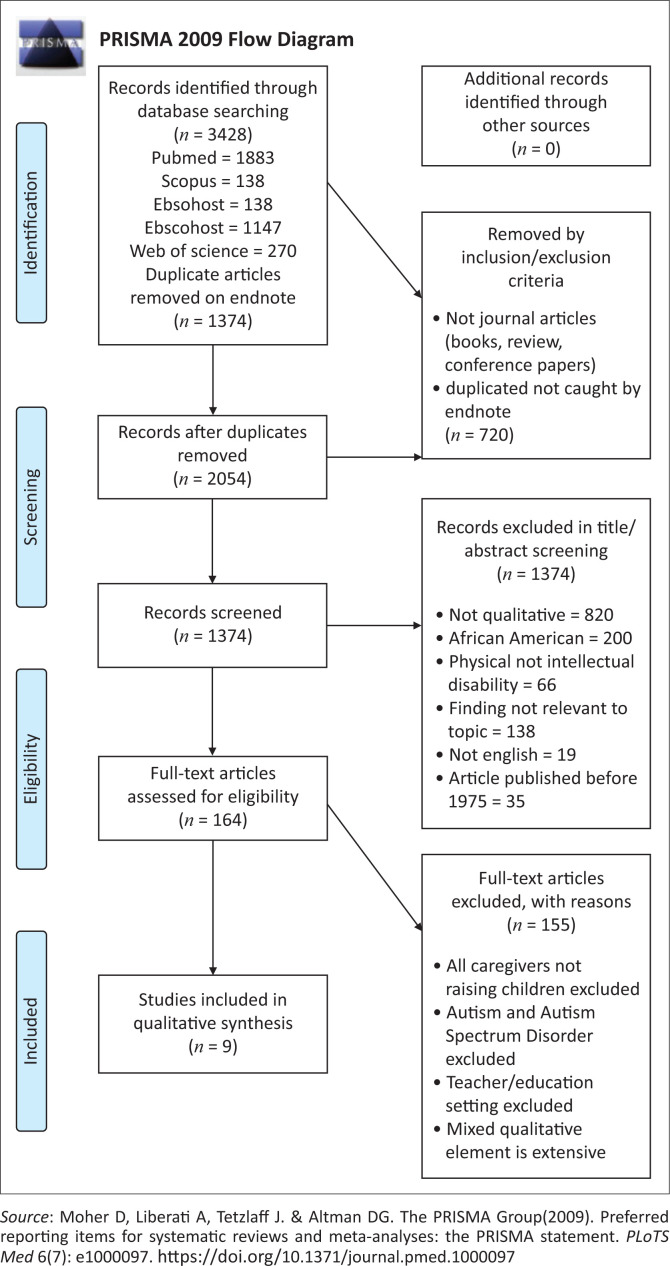
Prisma diagram.

There were initially 3428 articles identified through database searching (see Figure 1). After removing 1374 duplicates, 2054 articles remained and the abstracts perused. Of these, 720 articles, which clearly did not fall within our review’s area of concern, were removed based on exclusion criteria such as having been conducted on animal, not human, subjects and being conference papers, reviews, books and other grey literature, rather than journal articles. A further 1318 were then systematically removed during a process of screening abstract titles. Subsequently, 164 full articles were screened, leaving the final nine, which met all identified criteria.

### Eligibility criteria

Studies included in the review met the following criteria: (1) contained empirical research utilising qualitative research methodology, (2) focused on caregiver, parents or family experiences of raising a child with ID and (3) articles focused on African countries or Africa. Table 1 details the inclusion and exclusion criteria.

**TABLE 1:** Eligibility - inclusion and exclusion criteria.

Inclusion criteria	Exclusion criteria
Children and adolescents with ID	Studies on disability but not ID Studies on co-occurring presentations in which ID is not necessarily included by definition or syndromes in which ID is a variable outcome
Studies on caregivers, parents or family experiences of raising a child with ID	Studies on caregivers, parents or family experiences not raising a child with ID
Research sites and participants are in Africa	Study is not in Africa
Peer-reviewed academic journal articles	Study not in blind peer-reviewed journal (e.g. in a predatory journal)
Published between 1975 and 2018	Studies outside time limit
Full text available	Full text unavailable
English	Languages other than English

ID, intellectual disability.

### Data extraction

For full extraction of data, all duplicates were identified and removed. Following the removal of all duplicates, the remaining studies were screened for the full text. We then assessed the full text articles for eligibility by perusal of the abstracts. All full text articles that did not meet the eligibility criteria were excluded. All the articles that were obtained following assessment were then appraised by the three authors to ensure that they met the criteria for inclusion. All disagreements were resolved through discussion. Finally, the results from the eligible studies were systematically recorded on a summary sheet (see Table 2, for the articles extracted).

**TABLE 2:** Data extraction.

Authors	Topic	Study setting and country	Study design	Outcome
1. Ajuwon and Brown 2012	Family quality of life in Nigeria	Community agencies (Children’s Development Center and Open Doors for Special Learners), Nigeria	Mixed methods	Beliefs that influence behaviour and government policy and practice-Superstitious beliefs-Shopping for a cure-Parental attitude-Finance
2. Aldersey 2012	Family perceptions of intellectual disability: Understanding and support in Dar es Salaam	Participants’ homes, Dar es Salaam, Tanzania	Interviews, Grounded theory approach in a discussion	Families of people withintellectual disabilities. Search for meaning; Life after us; Whose responsibility? Something is wrong (Problems in diagnosis) Seeking a cure Acceptance Stigma What if we die? Employment Marriage Family The Tanzanian and international communities Government
3. Aldersey et al. 2014	Intellectual and developmental disabilities in Kinshasa, Democratic Republic of the Congo: Causality and implications for resilience and support	Parent self-help association, ANAPEH-MCO (L’Association Nationale des Parents des Enfants Vivant Avec Handicap Mentale en RDC), and through a research assistant, family homes and the wider community, Kinshasa, Democratic Republic of theCongo	Qualitative, narrative interviews	Mothers’ perceptions of the social needs of theiradolescent child; Mothers’ response to the social needs of theiradolescent child; Mothers’ perceptions of the emotional needs oftheir adolescent child; Mothers’ response to the emotional needs of their adolescent child; Mothers’ perceived support from professionals and other sources
4. Gona et al. 2011	Caring for children with disabilities in Kilifi, Kenya: What is the caregiver’s experience?	District hospital, homes and school, Kenya	Qualitative phenomenological approach	Challenges faced by caregivers: Shattered dreams; Expectations from healthcare staff. Coping strategies: Problem-focused; Emotion-focused.
5. Lamptey 2019	Health beliefs and behaviours of families towards the health needs of children with IDD in Accra, Ghana	Accra, Ghana	Qualitative, semi-structured interviews were the primary means of data collection	The study findings show that the influence of superstitious beliefs on the health beliefs and behaviours of families towards the health needs of children with IDD in Accra, Ghana, is mixed. The study findings highlight that families with such beliefs do not necessarily give up on medical care for the children.
6. Masulani-Mwale et al. 2016	Parenting children with intellectual disabilities in Malawi: the impact that reaches beyond coping?	Two clinics in two cities, Malawi	Qualitative phenomenological design and purposive sampling	Findings for this study are presented narratively in themes andsupported by verbatim quotes. Themes: -Challenges in care-Service inaccessibility-Stigma and discrimination-Impact on mental health-Coping and required supports
7. Nkhosi and Menon 2015	Mothers’ perceptions of the needs of adolescent children with intellectual disabilities at George Clinic, Lusaka, Zambia	Participants’ homes, Zambia	Qualitative method, focus group discussions and home observations	Discrimination Isolation Inadequate resources
8. Ntswane and Van Rhyn 2007	The life-world of mothers who care for mentally retarded children: the Katutura township experience	Participants’ homes, Namibia	Qualitative, explorative, descriptive and contextual design	Emotions: Acceptance and love; Feelings of despondency and sadness; Fear and shame; Anger and frustration; Worry.Relationships: Protectiveness; Rejection by spouse or partner.SocialCircumstances: Poverty and financial problems; Practical constraints in caring; Stigma.Physical needs: Problems of development.Support: Religion as spiritual support; Physical and emotional support; Bravery and defiance to stigma.
9. Tilahun et al. 2016	Stigma, explanatory models and unmet needs of caregivers of children with developmental disorders in a low-income African country: a cross-sectional facility-based survey	Two child mental health clinics, Ethiopia	Cross-sectional facility-based study, structured questionnaire, face-to-face interviews	High levels of stigma experienced by caregivers. Explanatory model of illness for caregivers. Interventions tried by caregivers. Unmet needs of caregivers. Coping strategies for caregivers. Gateway to the clinic.Limitations and strengths.

IDD, intellectual and developmental disabilities.

### Risk of bias

Nine studies met the inclusion criteria and were assessed for Risk of Bias (RoB). The Joanna Briggs Institute (JBI) Critical Appraisal Checklist for Qualitative Research (CAC) (Joanna Briggs Institute 2017) was used for this purpose. The CAC focuses on the design, conduct and analysis. The RoB assessments were administered to select studies for the analysis. S.M. conducted the RoB assessments and these were followed by discussions between S.M. and L.S. until consensus was reached. The details of quality assessment are provided in the supplementary material.

### Ethical considerations

Approval to conduct the study was obtained from Stellenbosch University REC: Humanities – reference number: REC-2017-0724.

## Results

### Characteristics of identified studies

The nine studies included in the review described the experiences of participants who have some form of relationship with, or have experience in caring for, a person with ID. Participants included families who have a family member or members with ID, caregivers and community members.

The study samples for all nine studies were attained from a wide range of sources. These included homes of the PWID, Children’s Development Centers, Open Doors for Special Learners, on the streets, special schools, vocational centres for people with intellectual developmental disorder (IDD), parental meetings and outings with the children.

With the exception of two studies, all studies were qualitative. The two exceptions were studies that applied mixed methods and offered sufficient qualitative data to be included in the final nine articles. Data collection consisted of interviews, questionnaires, in-depth interviews, standardised methods (e.g. Family Quality of Life Survey), observation methods and focus group discussions.

Using Braun and Clarke’s (2006) thematic analysis, we identified six themes (see Box 2) across the articles. These were understanding of ID, focussing on issues such as the meaning of ID, cultural beliefs, cure-seeking behaviour, stigma and discrimination; worries about the future, including death concerns, employment concerns, concerns about marriage and concerns about substitute caregivers; burden of care, referring largely to mental health concerns and depression in caregivers; lack of services, including gaps in education, health and social services; coping strategies and stigma and discrimination.

BOX 2 Major themes identified.

Understanding ID

Worries about the future

Burden of care

Lack of specialised ID services

Coping strategies

Stigma and discrimination

ID, intellectual disability.

### Quality ratings of identified articles based on the JBI Critical Appraisal Checklist for Qualitative Research

Assessment results based on CAC for Qualitative Research indicate that the quality of the nine studies was generally high. However, a source of possible bias was the lack of documented reflection on the possible influence of the researcher and the participants (see Table 2).

### Experiences of caregivers and parents living with children with intellectual disability

Nine studies were extracted and Table 2 shows an overview thereof. Next, these studies are discussed by theme.

#### Understanding and meaning of intellectual disability

Three studies sought to understand the causes and meaning participants gave to the diagnosis of ID (Aldersey 2012; Aldersey et al. 2014; Tilahun et al. 2016). All three studies looked at perceptions, understandings and explanations that caregivers and parents attribute to their children’s ID. Results show that various explanations were used in various communities across Africa depending on various cultural backgrounds. One qualitative study conducted participant observation and semi-structured interviews with family members of PWID and community members in Kinshasa, Republic of Congo (Aldersey et al. 2014), and reported that causes and meanings of ID in this population were founded on the belief that everything, including the occurrence of ID, happens for a reason. Reasons attributed to ID include superstition and mysticism, exemplified in the beliefs that the disability is a result of punishment from God, superstition about bewitchment and demon possession. The authors note that:

[*U*]nderstanding distinctions around the visible and invisible worlds is important in understanding the construction of meaning regarding ID in Kinshasa. In general, participants understood the causation of ID in biomedical (visible) or metaphysical (invisible) terms or a combination of both. (Aldersey et al. 2014:226)

Similarly, a study in Ethiopia, which utilised a mixed methodology approach to examining caregivers’ explanatory models of ID, reported that caregivers cited a combination of biomedical and supernatural factors as the cause of ID (Tilahun et al. 2016). Biomedical factors included head injuries, birth complications, pathogens, epilepsy and family history. Supernatural factors included the belief that ID was a form of punishment from God, demon possession and bewitchment. In this study, caregivers also admitted seeking cure from traditional practitioners as the first form of treatment following a diagnosis of ID, whilst others indicated seeking help from a biomedical practitioner, and across both groups, many had additionally sought help from other alternative sources such as religious healing centres, churches, priests and traditional healers (Tilahun et al. 2016).

#### Worries about the future

The future for the individual with ID was a common concern identified in some of the studies in the review (Ajuwon & Brown 2012; Aldersey et al. 2014; Gona et al. 2011). Future concerns varied from death of a primary caregiver, having one’s own family and finding employment. Death of a primary caregiver emerged as a major concern across the studies reviewed. Some studies perceived the importance of equipping individuals with ID with skills through education and training in order that PWID would be capable of looking after themselves should their caregiver pass away. Without these skills, caregivers reported that individuals with ID may struggle to contribute to meaningful social interactions (Nkhosi & Menon 2015). In addition, certain caregivers in some studies were concerned about who would replace them as primary caregivers should they die (Masulani-Mwale et al. 2016).

Furthermore, there were concerns about difficulty in finding employment for individuals with ID. These caregivers expressed disappointment that even post-school, the individual with ID would not be able to find work and gain independence, given the level of competency society expects of school-leavers (Gona et al. 2011).

#### Challenges of caregivers of children with intellectual disability: Burden of care

All reviewed studies explored challenges faced by caregivers of children with ID and reported that families experienced challenges with attaining support from services and from others or both. In particular, challenges were experienced with accessing disability and psychological services (Masulani-Mwale et al. 2016). Caregivers also talked about how an ID diagnosis shattered their dreams for their children (Gona et al. 2011). In the study by Tilahun et al. (2016), other challenges caregivers talked about included special needs educational services for their children, lack of treatment by a health professional, financial support to meet basic needs such as food and access to support from professionals in the management of a child with ID. Access to healthcare was also identified as a challenge by Lamptey (2019). Most of these studies reported that there is a significant lack of specialised education centres for children with ID and, in those countries, where they do exist, they are privately owned and very expensive. Caregivers and parents are then forced to resign from their jobs to provide full-time care for their children with ID. Other studies also reported a lack of specialised ID treatment services in some countries in Africa and these contributed to mental health difficulties experienced by both caregivers and children with ID themselves (Masulani-Mwale et al. 2016). In addition, one of the studies assessed quality of life for families of children with ID (Ajuwon & Brown 2012). Results from this study revealed that challenges experienced by caregivers and parents significantly compromised their quality of life.

Most caregivers in the studies reviewed expressed concerns about their own mental health. They described caring for an individual with special needs as being very stressful and, at times, traumatic. Most difficulties were attributed to the general presentation of the person with special needs. Problems were reported when there were challenging behaviours and a lack of basic skills (Masulani-Mwale et al. 2016; Nkhosi & Menon 2015). Types of challenging behaviours reported in these studies included physical aggression and inappropriate urination and defecating in public spaces. Caregivers reported that managing these difficulties was extremely stressful, evoking humiliation and embarrassment. Interestingly, none of the participants questioned the fact that the burden of care falls almost exclusively on women, and only one study (Masulani-Mwale et al. 2016) raised the issue of possible respite opportunities for caregivers.

#### Lack of specialised intellectual disability services

Concerns regarding the lack of specialised ID services for PWID and their caregivers presented as a significant concern for most participants in all studies reviewed, especially for those who expressed an interest in utilising such services. The review shows that PWID present with various difficulties requiring specialised clinical care from trained specialists. Although the caregivers’ help-seeking behaviours are generally determined by their belief systems, some participants in the reviewed studies expressed frustration regarding the lack of specialised services for PWID in their countries. These services include education, health and social services. In countries such as Nigeria, government policies on ID reportedly do not exist (Kagee et al. 2013). In two studies, participants who needed these services reported numerous attempts at trying to access government services without success (Masulani-Mwale et al. 2016; Nkhosi & Menon 2015). Data reveal that in some countries in Africa such services were terminated and were never established in others. In some countries, services such as specialised education or training are private and very expensive. As a result, most participants could not afford them. Participants also believed that they could benefit from specialised mental health services for themselves and for the individuals for whom they were caring, but these services were not available in their communities. Most participants reported that they needed mental health services, not only for their own psychological difficulties but also for their children’s behavioural and skills training. Most caregivers reported symptoms of depression and anxiety, which often overwhelm them and make it difficult to cope. For this, psychological services were identified as an urgent need to help them in dealing with these feelings.

#### Coping strategies

A number of studies reviewed described coping mechanisms of caregivers of children with ID. Findings from the majority of studies show that most participants used spirituality to cope with stress related to caring for a child with ID (Aldersey et al. 2014; Masulani-Mwale et al. 2016; Tilahun et al. 2016). Some studies in the review reported that most caregivers adopted spiritual interventions to cope with their situations. Relying on spiritual beliefs, some accepted that giving birth to, or caring for a child with, ID was God’s will. These caregivers would then take their children to churches to pray for deliverance. In addition, caregivers have reportedly used prayer as a coping mechanism even when at home. However, not all caregivers were fortunate enough to receive support from their churches; some were scared of going to church, fearing discrimination. Masulani-Mwale et al. (2016) reported on how some caregivers abandoned their faith because they were not fully accepted by their communities.

On the other hand, Gona et al. (2011) highlighted two coping strategies used by caregivers, with these strategies being problem focused and emotion focused. Caregivers reported empowering themselves by learning new home-based skills to better manage individuals with ID in their home environments. The authors describe how the caregivers trained their children to acquire basic skills such as walking and sitting. For them, this was necessary as access to professional services was scarce. Caregivers in this study also reported using emotion focused interventions to cope with the difficulties of caring for a child with ID. They indicated seeking spiritual support by going to church. Some also reported taking their children with ID to priests for deliverance. In addition, caregivers gathered together to share their experiences of caring for a child with special needs. Through this, they learned from each other’s experiences and advised each other on various issues. These findings were similar to those reported by Tilahun et al. (2016) who found that participant coping mechanisms included talking to a supportive adult and seeking religious guidance. For a minority of participants, coping involved the use of substances.

#### Stigma and discrimination

Most of the communities in which the studies in the reviews were conducted have proven to have very strong cultural beliefs. In most of the reviewed studies, caregivers and parents of PWID have reported being stigmatised because of others’ negative cultural beliefs and most of them reported having been subjected to high levels of stigma by their communities for caring for an individual with ID (Ajuwon & Brown 2012; Lamptey 2019; Masulani-Mwale et al. 2016; Tilahun et al. 2016). Some were called derogatory names and accused of intentionally causing their child’s ID as a way of gaining wealth because of their child’s disability (Masulani-Mwale et al. 2016). In addition, studies have reported that some communities within the African context perceive individuals with ID to be cursed or spirit possessed, resulting from sinful actions or punishment from God (Tilahun et al. 2016). In addition, Masulani-Mwale et al. (2016) found that certain caregivers and parents reported having been advised by some members of the community to kill their children with ID, advice which was rejected by the caregivers. Tilahun et al. (2016) further reported that participants worried ‘sometimes’, ‘often’ or ‘a lot’ about being treated differently. Furthermore, in this study, many participants worried about taking their child out of the house; felt ashamed or embarrassed about their child’s condition; felt a need to hide the problem from people in the community; made an effort to keep their child’s condition a secret and worried that people would be reluctant to marry into their family.

## Discussion

The results demonstrate both positive and negative experiences of those caring for PWID in Africa across all levels of the social system. The studies reveal the poignant reality of the daily struggles faced by PWID and their families.

In terms of Bronfenbrenner’s ecological framework, it is clear that caregivers and parents’ experiences are generally negative across a number of levels, from the micro-level of the family, through community and religious levels, to issues of care provision in African economies, all within the framework of global inequality. Themes associated with the micro-level included worries about the future and the burden of care having negatively affected their life experiences. Most participants across all nine studies expressed concerns about the future of their children with ID in the event that they should pass away. Others complained about not being supported by their extended family members, thus increasing the burden of care. Studies investigating parenting in the context of a family member with ID have reported similar findings where caregivers and parents of children with ID had to rely on their internal attributes, such as resilience, to cope rather than relying on others (Breitkreuz 2014).

In addition, at the micro-level, caregivers and parents struggled with mental health difficulties, including anxiety and stress, again in common with studies elsewhere. Perhaps more in the African context than elsewhere, given lack of access to resources and lack of custodial care, caregivers’ anxieties seem to be rooted in their perceived treatment by society, especially as this relates to society’s beliefs regarding the causes of ID. Lack of residential and day-care facilities result in PWID being cared for at home and in the community, thus unable to hide away from stigma, whereas some families, as in wealthier contexts, hide their family members with ID from the public eye (Aldersey et al. 2014; Haley & Perkins 2004; MacDonald & Hastings 2010).

Themes identified within the meso-system associated with services for PWID reveal the plight of caregivers and families of PWID in relation to accessing specialised services for their loved ones, as well as supportive services for themselves. The majority of studies reviewed have reported on the underdevelopment of ID and mental health services in Africa in general.

It is also clear from the articles we reviewed that the macro-system has a significant influence on caregivers’ and parents’ experiences of raising a child with ID. Most of the studies we reviewed reported on cultural or spiritual beliefs and the local resource context. There is a risk that beliefs that are viewed as superstitions are overemphasised in the literature, with more practical concerns being given less attention. In this regard, it is interesting that most recent of the articles reviewed (Lamptey 2019) open with a discussion of superstition and reliance on religious interventions, but the primary focus of the article is on the lack of access to resources, including healthcare resources. If people have not had access to all that biomedicine has to offer, then it is not surprising that their beliefs centre on explanations from other paradigms. In other words, what is presented as a difference in world view or belief system may in part be attributable to a difference in terms of access to resources.

In this regard, it is interesting that in the studies cited, there was a general understanding that the causation of ID was *both* supernatural *and* biomedical. It is clear that beliefs regarding the causes of ID, such as punishment from God, bewitchment, witchcraft and demon possession, do occur and need to be taken seriously; however, these are not the only views held in Africa.

These findings are consonant with those from previous studies from various parts of the world, showing, in multiple contexts, that there are a range of explanations for the causes of ID (Aldersey et al. 2014; Scior & Furnham 2011; Scior et al. 2015; Treloar 2002). Participants in the studies we reviewed used both western and indigenous health systems, but this is not a feature of African parents in particular – throughout the world, people seek to understand ID in a range of ways and may make use of a range of help, including services based on spiritual beliefs quite at odds with biomedical services (Sango & Forrester-Jones 2017).

Perhaps what is most striking, then, in our review, is not that parents relied on many systems of belief and help (this is in fact universal), but that the issues that parents and caregivers face are so similar to those reported in the literature in other parts of the world. The studies we reviewed showed evidence, which is common in the literature, of issues of shattered expectations, difficulties in adjustment and search for meaning in the context of the diagnosis of ID. What was strikingly different from the rest of the literature is the impoverished context of these parents and lack of access to the kinds of resources sometimes taken for granted elsewhere. Religion and spiritual beliefs are relied on heavily in this context, not necessarily because these are inherently more important here, but possibly because there is in reality little else on which people can rely.

An important implication of our study is that for the field of ID research to move forward globally, it is important to pay close attention to contextual and social factors. The experiences of caregivers in Africa are profoundly influenced by context and especially by lack of resources. For ID research to move forward, it is important to understand that the challenges faced by African parents are the rule rather than the exception in the global context.

The findings of this review present a number of practical implications for service provision in the field of ID. In particular, the review highlights the need for support services for caregivers of children with ID. Support services can be in the form of counselling and practical guidance from professionalised ID services. There is also a need to understand and leverage the informal forms of support caregivers identified as crucial support systems. These include spiritual and faith healers, prayer groups and churches. Finally, this review has identified the lack of specialised health, education and social services for PWID. It is clear from our study that much research is urgently needed on this topic, paying due regard to contextual issues and cross-cutting themes. Given the paucity of research, it is difficult to make arguments for policy changes, but accessibility of services and stigma are two clear emergent policy concerns.

A limitation of the review is that it does not provide a detailed in-depth analysis of the experiences of caregivers of children with ID, as there were very few studies, conducted in vastly different settings, employing different methodologies. However, it does provide an important starting point to understanding this topic within the African context. The paucity of research on this topic is a major problem; with hindsight, conducting a grey literature search on this topic might have provided a more in-depth analysis.

## Conclusion

A particular strength of this review is that it is the first of its kind in the African context, exploring caregivers’ experiences of raising and caring for a child with ID and thus addressing a gap in research into PWID and their caregivers. Findings of the review reveal that caregivers of children with ID in Africa face challenges regarding the lack of critically required specialised services for PWID in their countries. These services include education, health and social services. Findings indicate a need for formal and alternative healthcare sectors to work together in the understanding and management of ID in Africa. Furthermore, these findings raise important implications for research. Too few studies on ID have been conducted in the African context. Even fewer have been conducted on the particular experiences of those living with ID or caring for someone with ID. Only nine studies were suitable for inclusion in this review and even these studies did not demonstrate a uniform methodology, which is a key feature of a rigorous systematic review. Thus, a key implication for research is the need for more studies, particularly qualitative studies, to be conducted in the field of ID in different African contexts, exploring the role of culture, cultural beliefs, informal support systems and coping in the management of ID. What is also noteworthy is that despite the focus on cultural issues in the research that are on ID in Africa, there is relatively little engagement with questions related to the implications for African people of collectivist rather than individualist ideology and patterns of care. In disability studies more generally, there is increasing discussion of questions of Africanisation and decolonisation of knowledges (Mbazzi et al. 2020; Mji 2019; Owusu-Ansah & Mji 2013), but there has been less discussion of this in ID and family research. Future studies may well explore whether this is a fruitful line of research, helpful to children with ID in Africa and those who care for them.
